# Non-orthogonal one-step calibration method for robotized transcranial magnetic stimulation

**DOI:** 10.1186/s12938-018-0570-9

**Published:** 2018-10-01

**Authors:** He Wang, Jingna Jin, Xin Wang, Ying Li, Zhipeng Liu, Tao Yin

**Affiliations:** 1 Institute of Biomedical Engineering, Chinese Academy of Medical Science & Peking Union Medical College, Tianjin, 300192 China; 2 Neuroscience Center, Chinese Academy of Medical Science & Peking Union Medical College, Beijing, 100730 China

**Keywords:** Transcranial magnetic stimulation, TMS, Medical robots and systems, Hand/eye and coil calibration, Least-squares approach

## Abstract

**Background:**

Robotized transcranial magnetic stimulation (TMS) combines the benefits of neuro-navigation with automation and provides a precision brain stimulation method. Since the coil will normally remain unmounted between different clinical uses, hand/eye calibration and coil calibration are required before each experiment. Today, these two steps are still separate: hand/eye calibration is performed using methods proposed by Tsai/Lenz or Floris Ernst, and then the coil calibration is carried out based on the traditional TMS experimental step. The process is complex and time-consuming, and traditional coil calibration using a handheld probe is susceptible to greater calibration error.

**Methods:**

A novel one-step calibration method has been developed to confirm hand/eye and coil calibration results by formulating a matrix equation system and estimating its solution. Hand/eye calibration and coil calibration are performed to confirm the pose relationships of the marker/end effector ‘X’, probe/end effector ‘Y’, and robot/world ‘Z’. First, the coil is fixed on the end effector of the robot. During the one-step calibration process, a marker is mounted on the top of the coil and a calibration probe is fixed at the actual effective position of the coil. Next, the robot end effector is moved to a series of random positions ‘A’, the tracking data of marker ‘B’ and probe ‘C’ is obtained correspondingly. Then, a matrix equation system AX = ZB and AY = ZC can be acquired, and it is computed using a least-squares approach. Finally, the calibration probe is removed after calibration, while the marker remains fixed to the coil during the TMS experiment. The methods were evaluated based on simulation data and on experimental data from an optical tracking device. We compared our methods with two classical methods: the QR24 method proposed by Floris Ernst and the handheld coil calibration method.

**Results:**

The new methods outperform the QR24 method in the aspect of translational accuracy and performs similarly in the aspect of rotational accuracy, the total translational error decreased more than fifty percent. The new approach also outperforms traditional handheld coil calibration of navigated TMS systems, the total translational error decreased three- to fourfold, and the rotational error decreased six- to eightfold. Furthermore, the convergence speed is improved 16- to 27-fold for the new algorithms.

**Conclusion:**

These results suggest that the new method can be used for hand/eye and coil calibration of a robotized TMS system. Two complex steps can be simplified using a least-squares approach.

## Background

Transcranial magnetic stimulation (TMS) is a non-invasive and painless method for stimulating the cerebral cortex nerve [1–3]. Based on the principle of electromagnetic induction, an electric current is created on the cerebral cortex using a magnetic coil that is manually placed on top of the patient’s head. Recently, single-pulse and repetitive TMS have been used in clinical study for the therapy of mental disease [4–9]. However, TMS is still not widely promoted because its therapeutic effect changes between subjects [10, 11].

**Fig. 1:**
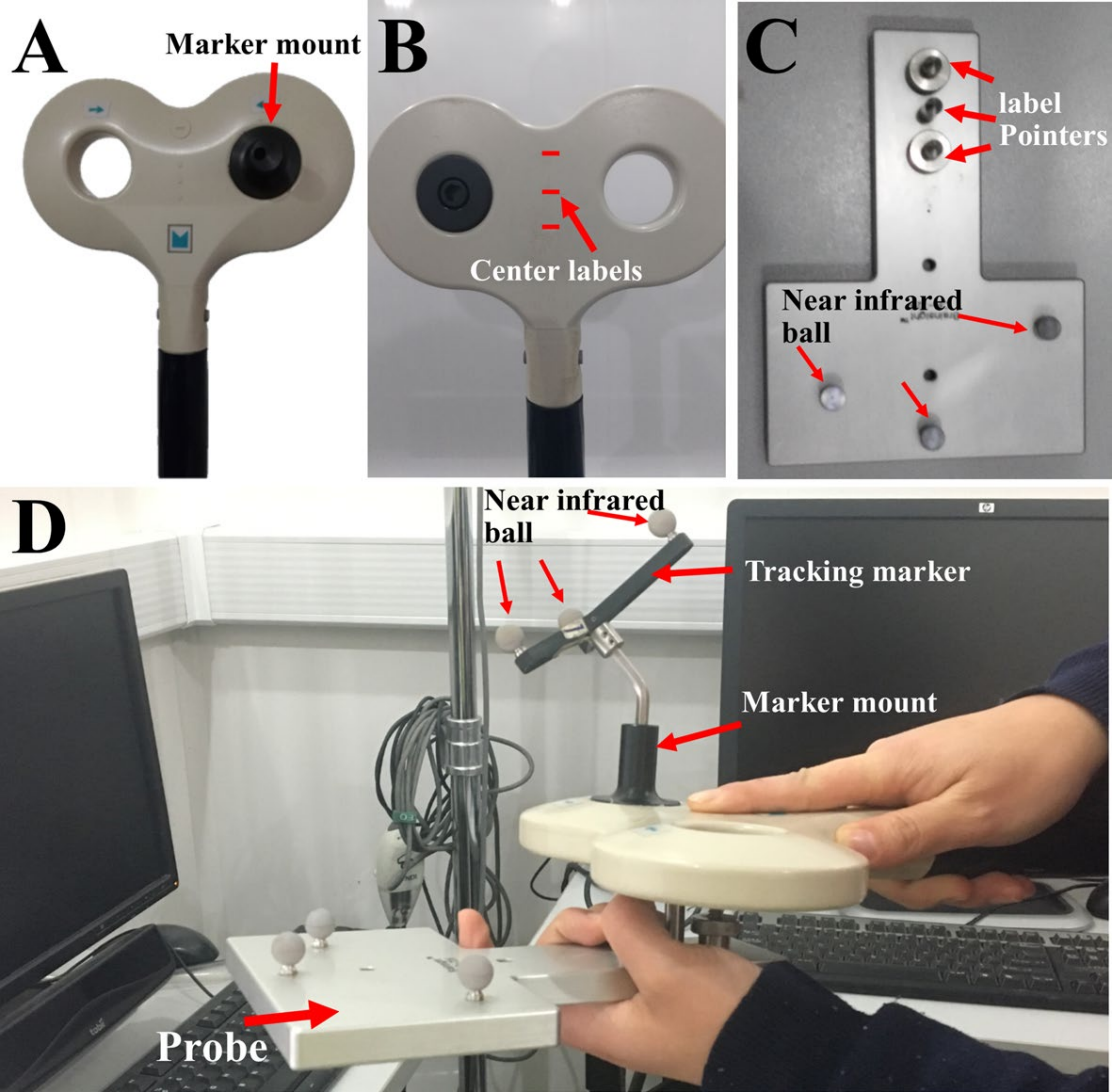
**A**, **B** Represent the top and bottom views, respectively, of a figure-of-eight stimulation coil (Rapid2, Magstim Inc., Whitland, Wales, UK); **C** the top view of probe (CB-725, BrainSight Inc., Montréal, Canada); **D** experimental picture of the coil calibration process

The variability is partly because of how accurate the stimulus process is carried out [12–14]. Initially, the magnetic stimulation coil was located over the subject’s head manually and imprecisely, without the help of any navigation system [10–12]. To solve this problem, neural-navigation technology has been developed to position the coil on the subject’s head more accurately [13, 14]. Magnetic resonance imaging (MRI) of both the subject and the optical tracking device are used in the neuro-navigation system, and navigation software can indicate the actual stimulation point of the coil on the subjects’ brain in real time. However, the stimulation coil, which usually weighs more than 2 kg (Fig. 1), is difficult for the operator to hold for more than 30 min in each procedure. Compared with handheld approach, a robot-assisted coil positioning method is more stable and repeatable, which is beneficial to the clinical or academic application of TMS.

Recently, two types of robotic TMS systems have been reported and shown to improve stimulation accuracy [15–18]. First, a robotic TMS system has been developed using a general industrial robot [15–17]. A six-jointed industrial robot (Adept Viper s850) was used in a robotic TMS system designed by Lars et al. [17]. For this kind of robotic system, the coil was mounted to the robot’s end effector, a Polaris Spectra infrared tracking system was used for navigation. After calibration, the magnetic coil is placed quite precisely and directly over a selected target region by the robot. However, the safety of this kind of robotic system has been queried, because the system is equipped with actuators selected for high-speed motions like any other industrial robot [18]. Second, a dedicated robotic system for TMS has been designed based on mechanical architecture and a control strategy [18]. A seven-jointed dedicated robot is designed from the kinematic scheme of the mechanism, including the arm, the prismatic joint, and the wrist [19]. The design and control of the robotic system optimizes the safety of the procedure, and allows the force applied between the robot and the head to be controlled. Robot-guided neuro-navigated TMS has been used in some recent studies of psychiatric diseases [19–21].

**Fig. 2:**
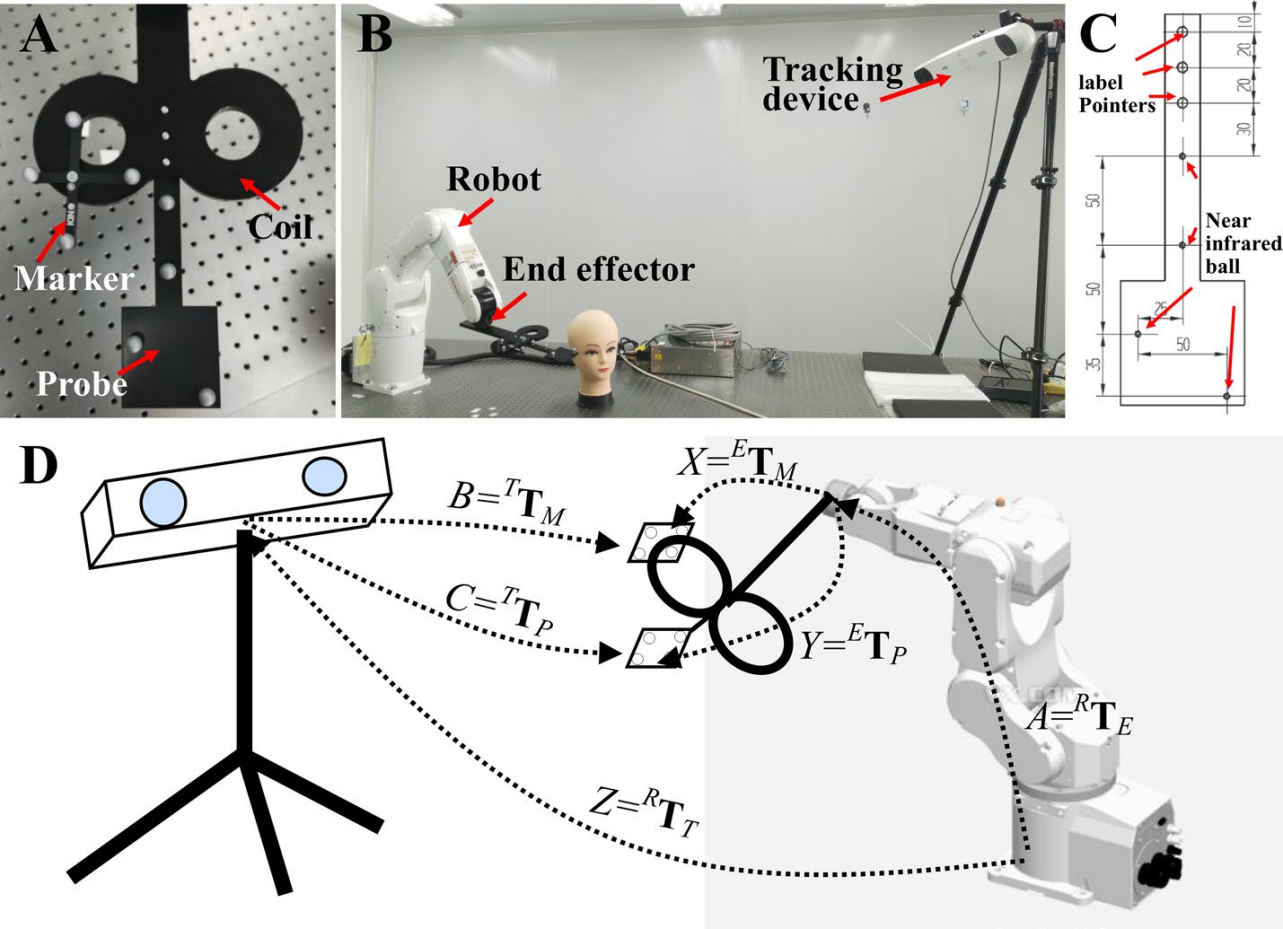
**A** The mechanical relationships among the coil, marker, and probe. **B** Set-up of the calibration experiment with an optical tracking device. **C** The mechanical drafting of the probe; the distance is marked in mm. **D** Principles of coil/end effector and robot/world calibration: a model of a figure-eight-coil is attached to the robot’s end effector E, a marker is fixed on the top of the stimulation coil, and a probe is fixed at the bottom of the coil

Robotized TMS systems are designed and requested for high-precision stimulus, which is primarily determined by calibration [17–22]. Besides, in order to ensure the safety of the robotic TMS system, a marker is mounted on the top of the coil to detect the coil position in real time [17–22], but the actual effective position of the coil is at the bottom centre of the coil, as shown in Fig. 1. Thus, hand/eye calibration and coil calibration are the most important steps in the workflow of a robotized TMS, and these two steps directly determine the accuracy of the system. Coil calibration involves confirming the first unknown pose matrices between the actual effective positions of the coil and the marker. Traditionally, a handheld coil calibration method is used, as shown in Fig. 1. A marker is fixed on the top of the coil (Fig. 1A), then a probe (Fig. 1C) is placed under the coil and the pointer is aimed at the coil centre labels (Fig. 1B, D). Finally, the positions of the marker and the coil centre in the coordinates of the tracking device can be detected, and the first unknown pose matrices can be calculated. Hand/eye calibration involves accurately computing the other two unknown pose matrices, which are the end-effector/marker matrix and the robot/tracking-device matrix [22, 23]. Traditional hand/eye calibration involves solving an AX = ZB form matrix equation, where the matrices ‘A’ and ‘B’ are known, and ‘X’ and ‘Z’ are unknown, as shown in Fig. 2D [22–26]. Typically, ‘A’ represents the transform from the robot’s base to the robot’s end effector, and ‘B’ represents the position and orientation data providing the full six degrees of freedom (DOF) of a marker obtained from a tracking device. ‘X’ represents the transforms from the robot’s end effector to the marker, and ‘Z’ represents the transform from the robot’s base to the tracking device.

The handheld coil calibration method typically suffers from larger calibration errors; a translational error of 3 mm is acceptable for handheld coil calibration [27–30]. In addition, the traditional hand/eye calibration method was reported separately by Shiu and Ahmad [23, 24] and Tsai and Lenz [25, 26]. Matrix algebra and the special properties of homogeneous matrices were used for determining the unknown matrices mentioned above. A review of these calibration algorithms was proposed by Wang et al. [31]. All these methods require that orthogonal homogeneous unknown matrices can be found. To overcome this limitation, the QR24 method, which uses three different variations on the basis of a naïve least-squares solution of the equation system, was developed for simultaneous hand/eye calibration [22]. However, all the hand/eye calibration methods mentioned above are used for determining two unknown pose matrices. For a robotized navigated TMS system, there are three unknown pose matrices, which need to be solved. The least-squares method can be applied to get unknown data and to minimize the error sum of squares between the gotten data and the actual data. For this reason, it is not unreasonable to assume that the simultaneous calibration of two steps in a naïve least-squares solution of the equation system might result in improved accuracy and efficiency.

In order to simplify the two complex calibration steps and improve the accuracy, this report presents an innovative approach for simultaneous hand/eye and coil calibration. A matrix equation system AX = ZB and AY = ZC was acquired at different robot positions, as shown in Fig. 2D. A linear equation system was obtained based on the matrix equation system, and a least-squares solution was calculated for determining ‘X’, ‘Y’, and ‘Z’. The feasibility and effectiveness of the method were demonstrated by comparing with two classical calibration methods: the QR24 method and the handheld coil calibration method.

## Methods

### Synchronous hand/eye and coil calibration

In this paper, we present a calibration method for robotized TMS with six DOF, which can be applied to any situation where there are three unknown pose matrices that need to be solved in the robotic system. As shown in Fig. 2A, B, typically, a coil is fixed to the end effector **E** of the robot **R**. A marker **M** with four near-infrared reflector balls is attached to the top of the coil and, synchronously, a probe **P** is fixed at the bottom centre of the coil. The mechanical drafting of the probe is shown in Fig. 2C. The tracking device **T** is placed so that the coil can be adjusted to the centre of the view of **T**. The design of this new approach to synchronous hand/eye and coil calibration is based on the following relationships:

1 $$ \begin{aligned}^{R}{\mathbf{T}}_{E}\;^{E}{\mathbf{T}}_{M}& =\,^{R}{\mathbf{T}}_{T}\;^{T}{\mathbf{T}}_{M} \\ ^{R}{\mathbf{T}}_{E}\;^{E}{\mathbf{T}}_{P} &=\,^{R}{\mathbf{T}}_{T} \;^{T}{\mathbf{T}}_{P} \\ \end{aligned} $$

where the matrix ‘^*R*^**T**_*E*_’ which is obtained by forward kinematic represents the transform from the robot’s base to the robot’s end effector, and the matrices ‘^*T*^**T**_*M*_’ and ‘^*T*^**T**_*P*_’ which are obtained directly from the tracking system represent the tracking data providing full six DOF of the marker and the probe, respectively. These three pose matrices are known parameters. The matrices ‘^*E*^**T**_*M*_’ and ‘^*E*^**T**_*P*_’ represent the transforms from the robot’s end effector to the marker and the probe, respectively, and ‘^*R*^**T**_*T*_’ represents the transform from the robot’s base to the tracking device. These three pose matrices are unknown parameters. To obtain the unknown parameters in Eq. 1, the end effector of the robot is moved to *n* different positions to obtain Eq. 2.

2 $$ \begin{array}{*{20}ll} {\left({^{R}{\mathbf{T}}_{E} } \right)_{i}^{E}{\mathbf{T}}_{M} =\;^{R}{\mathbf{T}}_{T} \left( {^{T}\mathbf{T}_{M} } \right)_{i} } \\ {\left({^{R}{\mathbf{T}}_{E} } \right)_{i}^{E}{\mathbf{T}}_{P} =\; ^{R}{\mathbf{T}}_{T} \left( {^{T}\mathbf{T}_{P} } \right)_{i} } \\ \end{array} \quad \begin{array}{*{20}l} {i = 1, \ldots ,n.} \\ \end{array} $$

Generally, the position of the robot’s end effector in Eq. 2 is chosen randomly in a sphere of radius r. The rotation angles of yaw, pitch, and roll for each position are also selected randomly between ± *d* degrees.

Let *A*_*i*_= (^*R*^**T**_*E*_)_i_, *X *= ^*E*^**T**_*M*_, *B*_*i*_ = (^*T*^**T**_*M*_)_i_, *Y* = ^*E*^**T**_*P*_, *C*_*i*_ = (^*T*^**T**_*P*_)_i_, and *Z* = ^*R*^**T**_*T*_. Then we obtain Eq. 3:

3 $$ \begin{array}{*{20}ll} {ZB_{i} - A_{i} X = 0} \\ {ZC_{i} - A_{i} Y = 0} \\ \end{array} \quad \begin{array}{*{20}l} {i = 1, \ldots ,n.} \\ \end{array} $$

Let

$$ w = \left[ {z_{1,1} ,z_{2,1} , \ldots z_{3,4} ,x_{1,1} ,x_{2,1} , \ldots x_{3,4} ,y_{1,1} ,y_{2,1} , \ldots y_{3,4} } \right]^{T} \in \mathbb{R}^{36}.$$

Thus, a linear system of equations can be derived from Eq. 3:

4 $$ Dw = b, $$

where $D \in \mathbb{R}^{24n \times 36} \;{\text{and }}b \in \mathbb{R}^{24n}.$ To be more specific,

5 $$ \begin{array}{*{20}ll} {D = \left[ {\begin{array}{*{20}ll} {D_{1} } \\ {D_{2} } \\ \vdots \\ {D_{n} } \\ \end{array} } \right]} \quad {\text{and}} \quad {b = \left[ {\begin{array}{*{20}ll} {b_{1} } \\ {b_{2} } \\ \vdots \\ {b_{n} } \\ \end{array} } \right]} \\ \end{array} $$

where

6 $$ \begin{array}{*{20}c} {D_{i} = \left[ {\begin{array}{*{20}c} {\begin{array}{*{20}c} {\text{R} [A_{i}^{ - 1} ]\left( {B_{i} } \right)_{1,1} } \quad {\text{R} [A_{i}^{ - 1} ]\left( {B_{i} } \right)_{2,1} } \quad {\text{R} [A_{i}^{ - 1} ]\left( {B_{i} } \right)_{3,1} } \quad {Zero_{3 * 3} } \\ {\text{R} [A_{i}^{ - 1} ]\left( {B_{i} } \right)_{1,2} } \quad {\text{R} [A_{i}^{ - 1} ]\left( {B_{i} } \right)_{2,2} } \quad {\text{R} [A_{i}^{ - 1} ]\left( {B_{i} } \right)_{3,2} } \quad {Zero_{3 * 3} } \\ {\text{R} [A_{i}^{ - 1} ]\left( {B_{i} } \right)_{1,3} } \quad {\text{R} [A_{i}^{ - 1} ]\left( {B_{i} } \right)_{2,3} } \quad {\text{R} [A_{i}^{ - 1} ]\left( {B_{i} } \right)_{3,3} } \quad {Zero_{3 * 3} } \\ {\text{R} [A_{i}^{ - 1} ]\left( {B_{i} } \right)_{1,4} } \quad {\text{R} [A_{i}^{ - 1} ]\left( {B_{i} } \right)_{2,4} } \quad {\text{R} [A_{i}^{ - 1} ]\left( {B_{i} } \right)_{3,4} } \quad {\text{R} [A_{i}^{ - 1} ]} \\ \end{array} } \quad { - Eye_{12} } \quad {Zero_{12} } \\ {\begin{array}{*{20}c} {\text{R} [A_{i}^{ - 1} ]\left( {C_{i} } \right)_{1,1} } \quad {\text{R} [A_{i}^{ - 1} ]\left( {C_{i} } \right)_{2,1} } \quad {\text{R} [A_{i}^{ - 1} ]\left( {C_{i} } \right)_{3,1} } \quad {Zero_{3 * 3} } \\ {\text{R} [A_{i}^{ - 1} ]\left( {C_{i} } \right)_{1,2} } \quad {\text{R} [A_{i}^{ - 1} ]\left( {C_{i} } \right)_{2,2} } \quad {\text{R} [A_{i}^{ - 1} ]\left( {C_{i} } \right)_{3,2} } \quad {Zero_{3 * 3} } \\ {\text{R} [A_{i}^{ - 1} ]\left( {C_{i} } \right)_{1,3} } \quad {\text{R} [A_{i}^{ - 1} ]\left( {C_{i} } \right)_{2,3} } \quad {\text{R} [A_{i}^{ - 1} ]\left( {C_{i} } \right)_{3,3} } \quad {Zero_{3 * 3} } \\ {\text{R} [A_{i}^{ - 1} ]\left( {C_{i} } \right)_{1,4} } \quad {\text{R} [A_{i}^{ - 1} ]\left( {C_{i} } \right)_{2,4} } \quad {\text{R} [A_{i}^{ - 1} ]\left( {C_{i} } \right)_{3,4} } \quad {\text{R} [A_{i}^{ - 1} ]} \\ \end{array} } \quad {Zero_{12} } \quad { - Eye_{12} } \\ \end{array} } \right]} \\ {{\text{and}},\quad b_{i} = \left[ {\begin{array}{*{20}c} {Zero_{9 * 1} } \\ { - T [A_{i}^{ - 1} ]} \\ {Zero_{9 * 1} } \\ { - T [A_{i}^{ - 1} ]} \\ \end{array} } \right]} \\ \end{array} . $$

The general form of the homogenous transformation matrix ‘*A*_*i*_^−1^’ can be presented as

$$  A_{i} ^{{ - 1}}  = \left[ {\begin{array}{*{20}c}    {{\text{R}}\left[ {A_{i} ^{{ - 1}} } \right]} & {T\left[ {A_{i} ^{{ - 1}} } \right]}  \\    0 & 1  \\   \end{array} } \right]  $$

where $\text{R} [A_{i}^{ - 1} ]\in {\mathbb{R}}^{ 3\times 3}$ represents the rotation matrix of ‘*A*_*i*_^−1^’ and $T [A_{i}^{ - 1} ]\in {\mathbb{R}}^{ 3}$ represents the translation vector of ‘*A*_*i*_^−1^’. ‘*Zero*_*m *× *n*_’ is a zero matrix of *m *× *n* size, and ‘*Eye*_*k*_’ is the identity matrix of *k *× *k* size. A least-squares method with QR-factorization can be used to solve a linear system of equations such as Eq. 4 [22]. In the definition of linear algebra, QR-factorization is a decomposition of the matrix ‘*D*’ into a result ‘*D *=* QR*’, where ‘*Q*’ is an orthogonal matrix and ‘*R*’ is an upper triangular matrix. All elements of unknown matrix ‘*X*’, ‘*Y*’ and ‘*Z*’ can be determined from the solution vector w of Eq. 4 based on the decomposed matrix ‘*Q*’ and ‘*R*’. All elements of unknown matrices ‘*X*’, ‘*Y*’ and ‘*Z*’ can be determined from the solution vector *w* of Eq. 4. It is important to point out that this method gives the optimum solutions for ‘*X*’, ‘*Y*’ and ‘*Z*’; thus, they minimize Eq. 7

7 $$ \sum\limits_{i = 1}^{n} {\left( {\left\| {A_{i} X - ZB_{i} } \right\|_{F} + \left\| {A_{i} Y - ZC_{i} } \right\|_{F} } \right)} $$

where ||*||_F_ is the Frobenius norm. The method presented above is called the QR36 calibration method, because a linear system of equations with 36 unknown parameters can be solved by QR-factorization.

To use these matrices, orthonormalization is the final and most important step, because ‘*X*’, ‘*Y*’ and ‘*Z*’ are not orthogonally solved in the QR36 method. Without this step, the homogeneous coordinate transformations obtained from ‘*X*’, ‘*Y*’ and ‘*Z*’ would be incorrect. In this paper, the singular value decomposition (SVD) method was used for orthonormalization. For a position N obtained from a tracking device, for which we want to calculate the position in robotic coordinates, we compute ZN to obtain a non-orthogonal matrix; next, the SVD of ZN is calculated as UΣV^T^ = ZN. Finally, the orthonormalized ZN can be calculated with (ZN)^⊥ ^= UV^T^.

### Calibration errors

With the calibration matrices ‘*X*’, ‘*Y*’ and ‘*Z*’ calculated by the QR36 method, we can obtain the calibration error using Eq. 1:

8 $$ \begin{aligned} E_{M} & = \left( {^{E}T_{M} } \right)^{ - 1} \left( {^{R}T_{E} } \right)^{ - 1}\;^{R}T_{T} \;^{T}T_{M} \\ E_{p} & = \left( {^{E}T_{P} } \right)^{ - 1} \left( {^{R}T_{E} } \right)^{ - 1} \;^{R}T_{T} \;^{T}T_{P}  \\ \end{aligned} $$

where ‘*E*_*M*_’ is defined as the calibration error matrix of the marker, and ‘*E*_*p*_’ is defined as the calibration error matrix of the probe. To obtain the calibration quality, we define the translational error and rotational error based on Eqs. 9–13. Let *O*_*M*_= *E*_*M*_^⊥^ and *O*_*p*_= *E*_*p*_^⊥^. Thus, the translational error of the system for QR36 method is defined as

9 $$ \begin{array}{*{20}ll}  e_{t} = e_{trans} [O_{p} ] + e_{trans} [O_{M} ]  \\ e_{trans} [O_{p} ] = \sqrt {{O_{p(1,4)}}^{2} + {O_{p(2,4)}}^{2} + {O_{p(3,4)}}^{2} }  \\ e_{trans} [O_{M} ] = \sqrt {{O_{M(1,4)}}^{2} + {O_{M(2,4)}}^{2} + {O_{M(3,4)}}^{2} }  \\  \end{array} \quad \begin{array}{*{20}c} {for} \quad {QR36} \\ \end{array}$$

where *e*_*t*_ is defined as the total translational error of the system, *e*_*trans*_[*O*_*p*_] is defined as the translational error of the probe, *e*_*trans*_[*O*_*M*_] is defined as the translational error of the marker.

Let (*k*_*M*_, *θ*_*M*_) and (*k*_*p*_, *θ*_*p*_) are the axis-angle representations of *R*(*O*_*M*_) and *R*(*O*_*p*_), respectively. Thus, the total rotational error of the system for QR36 method is defined as

10 $$ \begin{array}{*{20}ll} {\theta_{t} = \left| {\theta_{p} } \right| + \left| {\theta_{M} } \right|} \quad {for} \quad {QR36} \\ \end{array}.$$

where *θ*_*t*_ is defined as the total rotational error of the system, |*θ*_*p*_| is defined as the rotational error of the probe, |*θ*_*M*_| is defined as the rotational error of the marker.

Finally, if the marker and probe are calibrated separately using the QR24 method [22], then the calibration matrix *Z* will not be identical for both optimal calibration procedures. Thus, another translational error *e*_*z*_ is defined for the system:

11 $$ e_{z} = \sqrt {\left( {Z_{p(1,4)} - Z_{M(1,4)} } \right)^{2} + \left( {Z_{p(2,4)} - Z_{M(2,4)} } \right)^{2} + \left( {Z_{p(3,4)} - Z_{M(3,4)} } \right)^{2} } $$

where ‘*Z*_*p*_’ and ‘*Z*_*M*_’ are the calibration results of the probe and the marker calculated using the QR24 method. Thus, the translational error of the system for QR24 method is defined as

12 $$ \begin{array}{ll} e_{t} = e_{trans} \left[ {O_{p} } \right] + e_{trans} [O_{M} ] + e_{z}&  \\ e_{trans} \left[ {O_{p} } \right] = \sqrt {{O_{p(1,4)}}^{2} + {O_{p(2,4)}}^{2} + {O_{p(3,4)}}^{2} }  & \quad {for} \;{QR24}\\ e_{trans} \left[ {O_{M} } \right] = \sqrt {{O_{M(1,4)}}^{2} + {O_{M(2,4)}}^{2} + {O_{M(3,4)}}^{2} } & \end{array} . $$

where *e*_*t*_ is defined as the total translational error of the system, *e*_*trans*_[*O*_*p*_] is defined as the translational error of the probe, *e*_*trans*_[*O*_*M*_] is defined as the translational error of the marker.

Let (*k*_*z*_, *θ*_*z*_) be the axis-angle representation of *R*(*Z*_*p*_ − *Z*_*M*_); thus, the rotational error is defined as |*θ*_*z*_|. Thus, the rotational error of the system for QR24 method is defined as

13 $$ \begin{array}{*{20}ll} {\theta_{t} = \left| {\theta_{p} } \right| + \left| {\theta_{M} } \right|{ + }\left| {\theta_{z} } \right|} \quad {for} \quad {QR24} \\ \end{array}.$$

where *θ*_*t*_ is defined as the total rotational error of the system, |*θ*_*p*_| is defined as the rotational error of the probe, |*θ*_*M*_| is defined as the rotational error of the marker.

### Data acquisition

Simulated and optical tracking data were obtained to estimate the accuracy and robustness of the QR36 method. To compare the quality of our method with the QR24 and handheld methods, the QR24 method and the QR36 method were performed on the same dataset. The results are presented in the following section.

#### Simulated data

A set of simulated data was generated to test the proposed method [22]. Random orthogonal matrices X, Y, and Z with same definition in Eq. 3 were created first. To generate realistic values, the elements of T[X] and T[Y] were chosen randomly from [− 50, 50] mm, and the elements of T[Z] were chosen randomly from ±[500, 2000] mm. Subsequently, five hundred totally random orthogonal robot positions matrix *A*_*i*_ were created and used to calculate the corresponding tracking matrices *B*_*i*_ and *C*_*i*_. To get the simulated data of an optical tracking device, a marker and a probe with four markers was defined as

$$ marker = \left[ {\begin{array}{*{20}ll} 0 &{50} & {50} & 0 \\ 0 & 0 & {50} & {50} \\ 0 & 0 & 0 & 0 \\ 1 & 1 & 1 & 1 \\ \end{array} } \right],\quad probe = \left[ {\begin{array}{*{20}c} 0 & {25} & {25} & 0 \\ 0 & 0 & {25} & {25} \\ 0 & 0 & 0 & 0 \\ 1 & 1 & 1 & 1 \\ \end{array} } \right]. $$

In order to distort the tracking matrices $\tilde{B}_{i}$ and $\tilde{C}_{i} ,$ the marker and the probe were moved to the pose described by *B*_*i*_ and *C*_*i*_, and according to Horn’s algorithm, the matrix was computed without scaling [26], that is,

$$ \begin{aligned} \tilde{B}_{i} & = horn\left( {marker,\;B_{i} *marker + \sum_{i} } \right) \\ \tilde{C}_{i} & = horn\left( {probe,\;C_{i} *probe + \sum_{i} } \right) \\ \end{aligned} $$

Here,

$$ \sum\nolimits_{i} { = }\left[ {\begin{array}{*{20}ll} {s_{i,1,1} } & {s_{i,1,2} } & {s_{i,1,3} } & {s_{i,1,4} } \\ {s_{i,2,1} } & {s_{i,2,2} } & {s_{i,2,3} } & {s_{i,2,4} } \\ {s_{i,3,1} } & {s_{i,3,2} } & {s_{i,3,3} } & {s_{i,3,4} } \\ 0 & 0 & 0 & 0 \\ \end{array} } \right] $$

where *s*_*i*, *j*, *k*_ was obtained from the Gaussian distribution with standard deviation equal to 0.05 mm.

A different method was used to distort the robot matrices: the rotational matrix was distorted by multiplication with a rotation matrix around a random axis *a*_i_. The rotation angles *θ*_*i*_ was drawn from the Gaussian process with standard deviation equal to 0.05 mm. Gaussian noise was used to distort the translational vector:

$$ \tilde{A}_{i} { = }\left[ {\begin{array}{*{20}ll} {\text{R}[A_{i} ]*rot_{{a_{i} }} (\theta_{i} )} & {T[A_{i} ] + \sum_{i} } \\ 0 & 1 \\ \end{array} } \right] $$

where

$$\sum\nolimits_{i}  = \left[ {\begin{array}{*{20}ll} {s_{i,1} } & {s_{i,2} } & {s_{i,3} } \\ \end{array} } \right]^{T} $$

*s*_*i*, *k*_ was obtained from the Gaussian distribution with standard deviation equal to 0.05 mm.

#### Data from optical tracking device

As shown in Fig. 2B, C, an optical tracking device (Polaris Spectra, Northern Digital Inc., Waterloo, Ontario, Canada) was mounted on tripods, and a coil model was fixed at the end effector of a 6-axis robot (VS060, Denso Inc., Aichi, Japan). The marker was attached to the top of the coil, and the probe was fixed at the bottom centre of the coil. Then, the position of the end effector and the tracking device were adjusted so that the positions of the marker and probe were near the centre of the tracking device’s work volume (that is, |x| < 100 mm, |Y| < 100 mm, and Z = − 1500 ± 100 mm for both the marker and the probe), and the position of the end effector was defined as first position *P*_0_. Next, the end effector was moved to 500 random positions around the first position *P*_0_. The positions were confirmed by

14 $$ P_{i} = P_{0} *T(t)*R_{x} (\theta_{1} )*R_{y} (\theta_{2} )*R_{z} (\theta_{3} ), $$

where *R*_*x*, *y* and *z*_ (θ) is a rotational matrix around the x, y and z-axis, in which θ was randomly selected from − 10° to 10°, and T is a translation matrix with translation vector *t* that was randomly selected in the range from − 100 to 100 mm. 100 measurements (*A*, *B*, *C*) were averaged to reduce the errors from the robot and optical tracking device.

## Results

### Evaluation and implementation

The algorithms were evaluated on a standard personal computer (Intel core E7500 CPU with 4 GB of RAM, running 64-bit Windows 10 OS). The algorithms were all implemented in MATLAB 2010a. The performance period was less than 0.1 s for the QR24 and QR36 algorithms.

### Calibration errors

The QR36 algorithm was performed using poses *P*_1_,…,*P*_*n*_ for *n *= 5,…,250. The rotational and translational errors were computed for each remaining testing pose *P*_251_,…,*P*_500_ using Eqs. 9 and 10, and the average of all 250 testing poses was determined. The QR24 algorithm was calculated on the marker and the probe separately using poses *P*_1_,…,*P*_*n*_ for *n *= 5,…,250. The rotational and translational errors of the marker and the probe were computed for each remaining testing pose *P*_251_,…,*P*_500_ using Eqs. 12 and 13, and the average of all 250 testing poses was determined. Then, error *E*_*z*_ and |*θ*_*z*_| were determined for each remaining testing pose *P*_251_,…,*P*_500_, and the average of all 250 testing poses was determined.

### Simulated results

**Fig. 3:**
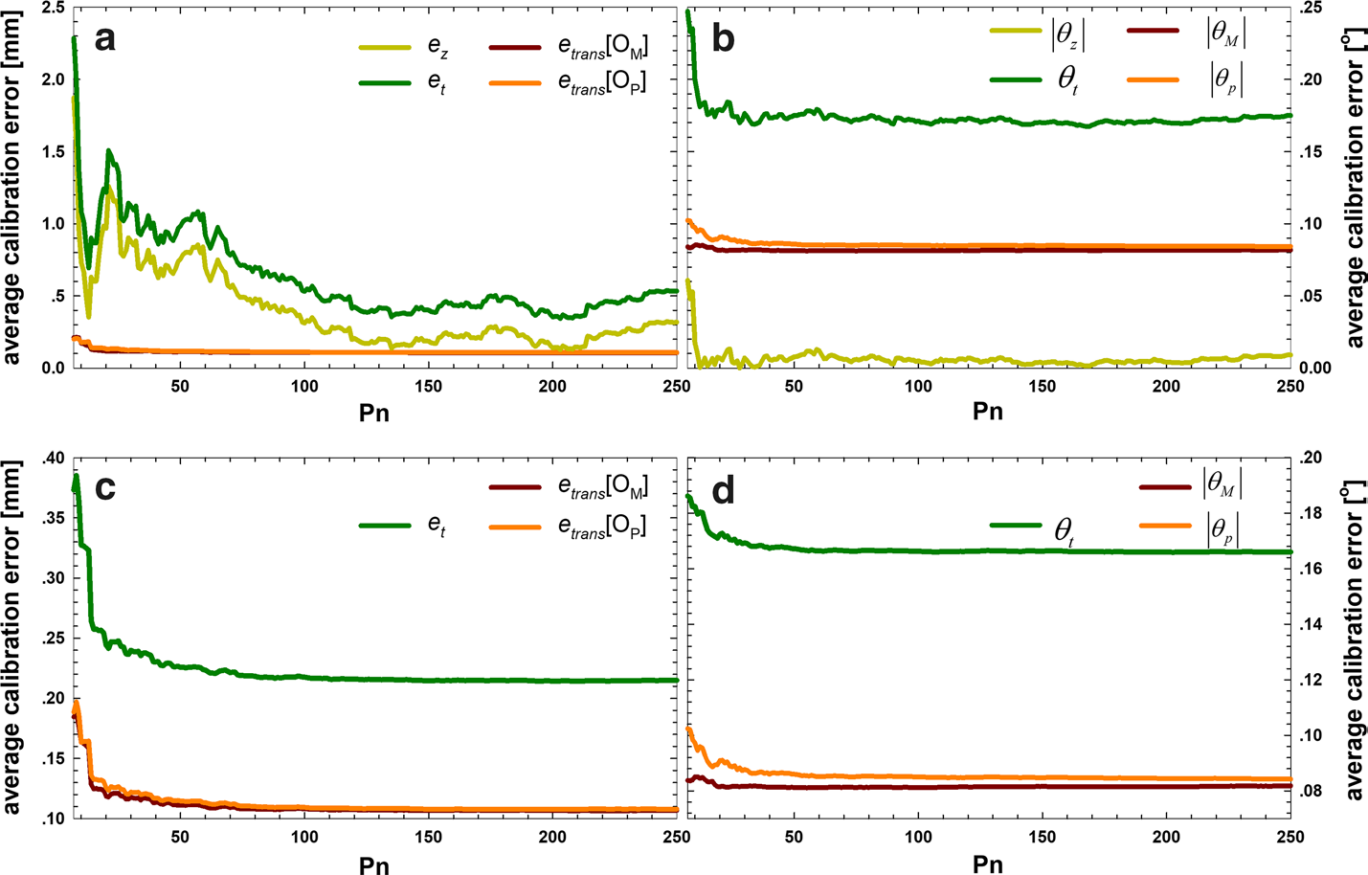
Calibration errors for the QR24 and QR36 algorithms with the simulated data. The algorithms used *n *= 5,…,250 poses to compute the calibration matrices that were tested on 250 remaining poses. **a** Translational error for the QR24 method, **b** rotational error for the QR24 method, **c** translational error for the QR36 method, and **d** rotational error for the QR36 method

Figure 3 presents the average errors for the simulated data using the QR24 method (top) and the QR36 method (bottom).

Clearly, using more than 20 poses hardly influences the translational and rotational errors of the QR36 method. However, using the QR24 method to calibrate the marker and probe separately could create two different calibration matrices Z. This can create a new calibration error *e*_*z*_, which significantly influences the translational error of the QR24 algorithm. In the simulated data, the resulting error came very close to the error obtained using the correct matrices, and remained within 1%. The minimum total translational error decreased 59.54% for the simulated data. The minimum total translational error *e*_*t*_ of the QR24 method was 0.3419 mm, with an *e*_*z*_ of 0.1267 mm, while the minimum total error of the QR36 method was 0.2143 mm. In other words, without error *e*_*z*_, the minimum total errors of the QR24 method and the QR36 method are approximately equal; the same result was obtained from the data for the 25th, median, and 75th errors, but not the maximum total error. Insufficient sample data produced an extremely large error *e*_*z*_; therefore, our method is more useful for a small data set. Overall, both convergence speed and precision of the translational error were improved for the QR36 method compared with the QR24 method. However, although the QR36 method outperformed the QR24 method regarding the rotational error of *θ*_*t*_, the effect of *θ*_*z*_ was very slight. The *θ*_*z*_ of the QR24 method only had a 0.78% influence on the minimum total rotational error.

Additionally, Table 1 presents the statistics of the errors for the simulated data shown in Fig. 3. The minimum, 25th, median, 75th, and maximum total translational error *e*_*t*_ and total rotational error *θ*_*t*_ and corresponding parameters are summarized in Table 1.

**Table 1:** Error statistics of the calibration algorithms using matrices on the simulated data

Alg	Para	Min	25th	Med	75th	Max
QR24	Translational error (mm)					
	* e*_*trans*_[*O*_*p*_]	0.1071	0.1075	0.1074	0.1088	0.5134
	* e*_*trans*_[*O*_*M*_]	0.1081	0.1085	0.1081	0.1138	0.7211
	* e*_*z*_	0.1267	0.2059	0.2735	0.5078	5.9305
	* e*_*t*_	0.3419	0.4219	0.4889	0.7305	7.1649
	* P*_*n*_	203	147	182	73	6
	Rotational error (°)					
	* θ*_*p*_	0.0814	0.0814	0.0812	0.0818	0.0839
	* θ*_*m*_	0.0847	0.0847	0.0851	0.0842	0.1024
	* θ*_*z*_	0.0013	0.0043	0.0056	0.0079	0.0609
	* θ*_*t*_	0.1675	0.1704	0.1719	0.1739	0.2472
	* P*_*n*_	168	173	97	245	7
QR36	Translational error (mm)					
	* e*_*trans*_[*O*_*p*_]	0.1066	0.1068	0.1071	0.1088	0.3004
	* e*_*trans*_[*O*_*M*_]	0.1076	0.1079	0.1084	0.1118	0.3203
	* e*_*z*_	0	0	0	0	0
	* e*_*t*_	0.2143	0.2148	0.2155	0.2206	0.6207
	* P*_*n*_	202	158	133	64	6
	Rotational error (°)					
	* θ*_*p*_	0.0815	0.0812	0.0815	0.0813	0.0861
	* θ*_*m*_	0.0844	0.0848	0.0848	0.0853	0.1064
	* θ*_*z*_	0	0	0	0	0
	* θ*_*t*_	0.1659	0.1661	0.1663	0.1665	0.1925
	* P*_*n*_	203	107	142	69	6

### Experimental results

**Fig. 4:**
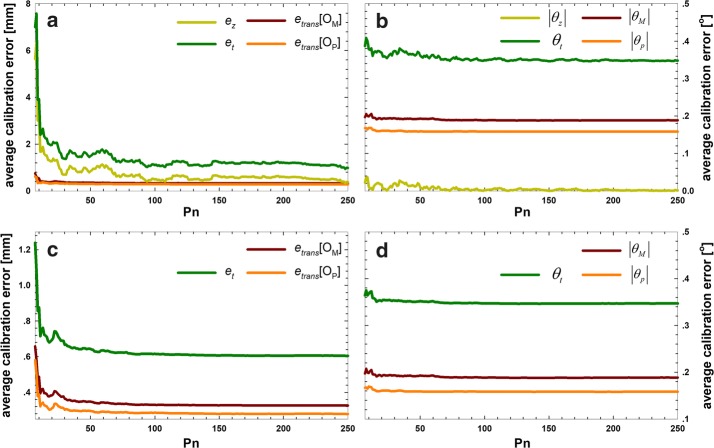
Calibration errors for the QR24 and QR36 algorithms with the experimental data. The algorithms used *n *= 5,…,250 poses to compute the calibration matrices that were tested on 250 remaining poses. **a** Translational error for the QR24 method, **b** rotational error for the QR24 method, **c** translational error for the QR36 method, and **d** rotational error for the QR36 method

Figure 4 presents the average errors for the optical tracking device. We obtained similar results from the experimental data. The translational errors of the QR24 and QR36 methods were hardly changed by using more than 30 poses. In the experimental data, the resulting error became much larger than the error obtained from simulation. We also found that the error *e*_*z*_ had a greater influence on the translational error of the experimental data. The minimum total translational errors were decreased by 54.93%, as 0.9353 mm and 0.6037 mm for QR24 and QR36, respectively. *e*_*z*_ had a minimum total translational error of 0.3301 mm. The effect of *θ*_*z*_ on the rotational error of *θ*_*t*_ was very small, according to the experimental data. The maximum total translational errors were 8.3719 and 1.2384 mm for QR24 (*P*_*n*_= 5) and QR36 (*P*_*n*_= 6), respectively. *e*_*z*_ had a translational error of 5.7679 mm, indicating that fewer tracking data will lead to a larger calibration error *e*_*z*_.

Additionally, Table 2 presents the statistics of the errors for the experimental data shown in Fig. 4. The minimum, 25th, median, 75th, and maximum total translational error *e*_*t*_ and total rotational error *θ*_*t*_ and corresponding parameters are summarized in Table 2.

**Table 2:** Error statistics of the calibration algorithms using matrices on the experimental data

Alg	Para	Min	25th	Med	75th	Max
QR24	Translational error (mm)					
	* e*_*trans*_[*O*_*p*_]	0.3263	0.3296	0.3272	0.3331	1.4110
	* e*_*trans*_[*O*_*M*_]	0.2787	0.2845	0.2787	0.2854	1.1929
	* e*_*z*_	0.3301	0.5024	0.5935	0.6995	5.7679
	* e*_*t*_	0.9353	1.1166	1.1995	1.3181	8.3719
	* P*_*n*_	247	109	200	81	5
	Rotational error (°)					
	* θ*_*p*_	0.1882	0.1889	0.1886	0.1883	0.2157
	* θ*_*m*_	0.1584	0.1590	0.1583	0.1583	0.1825
	* θ*_*z*_	0.0005	0.0003	0.0030	0.0069	0.0832
	* θ*_*t*_	0.3466	0.3482	0.3500	0.3536	0.4815
	* P*_*n*_	176	88	190	127	5
QR36	Translational error (mm)					
	* e*_*trans*_[*O*_*p*_]	0.3256	0.3261	0.3277	0.3353	0.6580
	* e*_*trans*_[*O*_*M*_]	0.2781	0.2797	0.2807	0.2873	0.5803
	* e*_*z*_	0	0	0	0	0
	* e*_*t*_	0.6037	0.6059	0.6084	0.6226	1.2384
	* P*_*n*_	250	227	132	73	6
	Rotational error (°)					
	* θ*_*p*_	0.1880	0.1883	0.1885	0.1893	0.2074
	* θ*_*m*_	0.1583	0.1584	0.1584	0.1586	0.1674
	* θ*_*z*_	0	0	0	0	0
	* θ*_*t*_	0.3464	0.3467	0.3470	0.3480	0.3749
	* P*_*n*_	141	128	217	68	7

Convergence properties. To acquire the convergence properties of the two algorithms, the simulated and experimental data were assessed again. It is clear from Figs. 3 and 4 that the QR36 method converged faster than the QR24 method. Additionally, it was possible to determine whether and how fast the algorithms converged to set values, which were presented in this paragraph. The results are shown in Table 3. Again, we can see that the convergence speed is improved 16- to 27-fold for the QR36 algorithms, stabilizing at a translational error of less than 0.4 mm and 0.3 mm at *P*_*n*_= 7 and 14, respectively. For the QR24 method, translational errors of < 0.4 and 0.3 mm were found at *P*_*n*_= 118 and none, respectively. The translational errors of the experimental data followed a similar pattern: the convergence properties of the QR36 method were much better than those of the QR24 method. However, the rotational errors showed a different picture: the accuracy of less than some set values is achieved on both algorithms similarly.

**Table 3:** Convergence properties of the calibration algorithms using matrices on the simulated and experimental data

Data group	Algorithm	Error type	Total errors	*P*_*n*_
Simulation	QR24	Translation	< 0.4 mm	118
			< 0.3 mm	None
		Rotation	< 0.2°	10
			< 0.17°	28
	QR36	Translation	< 0.4 mm	7
			< 0.3 mm	14
		Rotation	< 0.2°	6
			< 0.17°	22
Experiment	QR24	Translation	< 1 mm	247
			< 0.7 mm	None
		Rotation	< 0.4°	10
			< 0.35°	66
	QR36	Translation	< 1 mm	9
			< 0.7 mm	27
		Rotation	< 0.4°	6
			< 0.35°	57

#### Workspace sizes

**Fig. 5:**
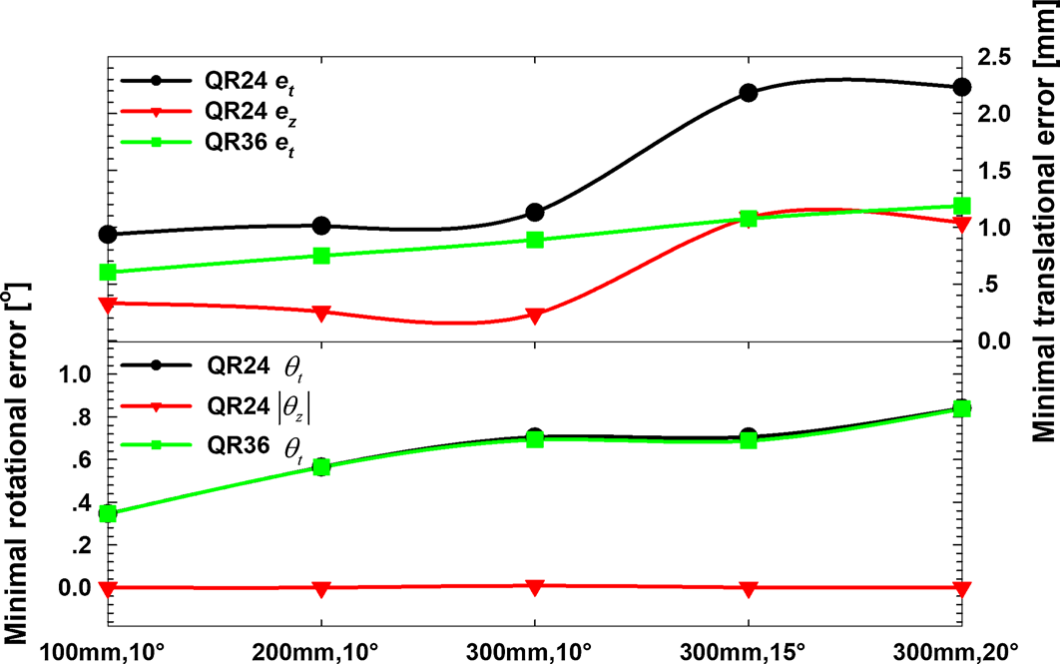
Influence of the changes in the size of the calibration workspace on the best calibration results achievable for the QR24 and QR36 algorithms

Workspace sizes have a substantial influence on the calibration error in many medical robotic applications. To demonstrate the quality of our method in this regard, additional calibration experiments with the Polaris Spectra system and increased workspace sizes were performed. Five different workspace sizes were chosen for the experiments (100 mm, 10°; 200 mm, 10°; 300 mm, 10°; 300 mm, 15°; and 300 mm, 20°). In all cases, 500 samples were successfully recorded. The minimum errors for different workspace sizes are presented in Fig. 5. Interestingly, the total translational error of the QR36 method increased with workspace size. However, the total translational error of the QR24 method was influenced by error *e*_*z*_. *e*_*z*_ did not increase linearly with increasing workspace size; however, it did create a smaller calibration error for a large workspace (*e*_*z*_= 1.04 mm for a workspace of 300 mm, 20°). The total rotational error of the QR24 and QR36 methods all increased with increasing workspace size, but error *e*_*z*_ had a small influence.

### Handheld coil calibration

To compare the quality of our method with the handheld coil calibration method, two operators with similar experience of TMS participated in this experiment. The QR36 method and handheld calibration method were performed by the two operators. The translational error *e*_*trans*_[*O*_*p*_] and rotational error *θ*_*p*_ of the probe, which were defined in Eqs. 9 and 10, were estimated to evaluate the calibration accuracy.

A set of five additional data acquisition experiments using the robotic TMS system in a workspace of 300 mm (10°) were performed by each operator. For the experimental procedure, refer to the experimental data acquisition paragraph in the Methods section. For each experiment, the probe was remounted on the coil by the operator, and 500 measurements (*A*_*i*_, *B*_*i*_, *C*_*i*__i = 1,…,500_) were collected, with the end effector moving to 500 random positions around the first position *P*_0_. Next, the robot was moved to the first position P_0_, and the probe was unloaded from the coil and held at the bottom centre labels of the coil by the operator. An additional measurement (*B*_*a*_, *C*_*a*_) was acquired with an optical tracking device. The acquired 500 measurements and the additional measurement were defined as the dataset of each experiment.

**Fig. 6:**
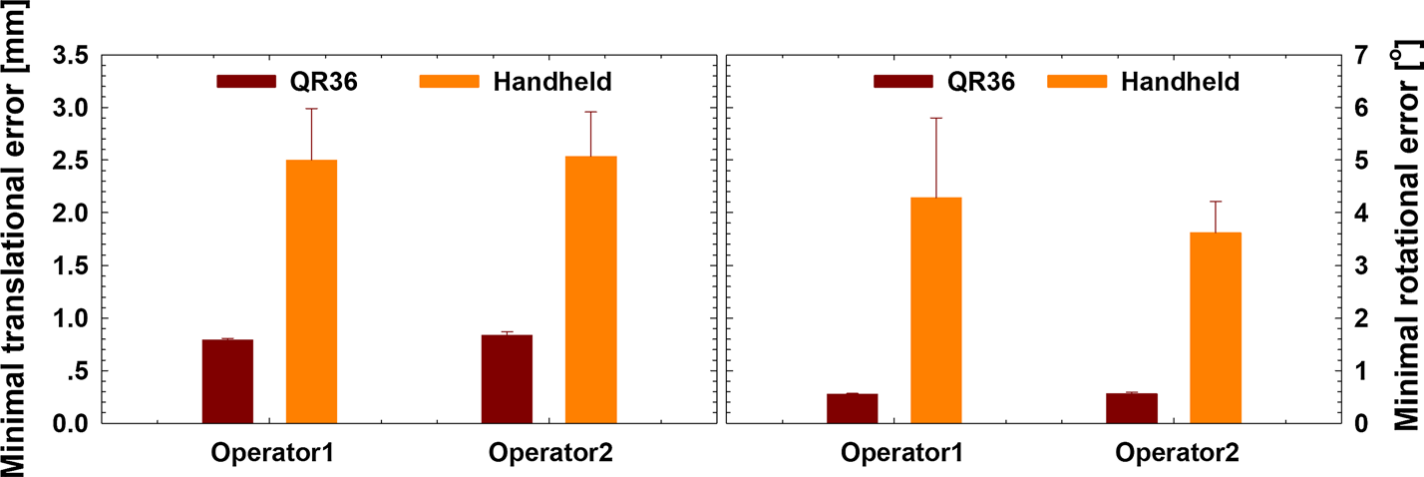
Calibration errors of the QR36 method and the handheld coil calibration method for different operators. The symbols and error bars represent the mean values and the standard deviations, respectively, obtained from five optimal errors via five separate experiments

The QR36 algorithm was performed using poses *P*_1_,…,*P*_*n*_ for n = 5,…,250 of each experiment. The rotational and translational errors of the probe were computed for each remaining testing pose *P*_251_,…,*P*_500_ using Eqs. 9 and 10, and the average of all 250 testing poses was determined. Then, the optimal translational and rotational calibration errors (MIN (*e*_*trans*_[*O*_*p*_])_n_ and MIN(*θ*_*p*_)_n_ for n = 5,…,250) were determined for each experiment. Finally, the mean value and standard deviation of the optimal calibration errors for five experiments per operator were determined. The results are presented in Fig. 6.

The traditional separate two-step calibration with QR24 and the handheld method were carried out for comparison with the QR36 method. Based on the dataset of marker (*A*_*i*_, *C*_*i*__i = 1,…,500_), the optimal calibration result (X, Z) was determined by the QR24 method with the minimal translational error. Subsequently, Y was calculated using Eq. 13 with the additional position pair (*B*_*a*_, *C*_*a*_) [24]. For each experiment, the translational error *e*_*trans*_[*O*_*p*_] and rotational error *θ*_*p*_ of the probe were computed for each testing pose (*B*_*i*_, *C*_*i*__i = 251,…,500_) with the calibration result (Y, Z), and the average of all 250 testing poses was determined as the handheld calibration error of the experiment. Finally, the mean values and standard deviations of calibration errors of five experiments for each operator were determined. The results are presented in Fig. 6. All the symbols (A, B, C, X, Y, Z) in this section are as defined in Eq. 3.

15 $$ Y = {XB_{a}}^{ - 1} C_{a}.$$

As shown in Fig. 6, both the mean values and the standard deviations of the rotational and translational errors significantly decreased when the QR36 method was used; the total translational error decreased three- to fourfold, and the rotational error decreased six- to eightfold. For the two operators, the minimum total translational errors for QR36 method were decreased to 0.7931 and 0.8361 mm, respectively, as compared with 2.4997 and 2.5354 mm for handheld method. The minimum total rotational errors were decreased to 0.5509° and 0.5624° using QR36 method, as compared with 4.2808° and 3.6202° using handheld method. The rotational error showed greater improvement, probably because there was still a positioning label on the coil for the position probe, whereas there is no method for reducing the perspective error of the operator. Moreover, because the probe was fixed on the coil mechanically and an optimization algorithm was used, no significant differences in either the translational or the rotational errors of the QR36 method were found for different operators. Finally, the QR36 method showed better robustness to choice of operator than the handheld coil calibration method.

## Discussion

An innovative approach for synchronous hand/eye and coil calibration has been presented and evaluated. A least-squares method that has been widely used in medical imaging and robotics was used to estimate the optimal calibration matrix [32, 33]. The approach has been validated with synthetic data and experimental data from an optical tracking device. The calibration effect of our method was compared with that of traditional methods such as QR24 and the handheld coil calibration method.

These results show that QR36 calibration method is advisable for use in the robotized TMS system. We have shown that both the QR24 method and our new QR36 method perform very well, with errors below 1 mm and 0.2°, using both simulated and experimental data. This shows that the two methods can both be used for typical calibration. However, whereas more than 200 different robot positions were required to achieve a calibration error below 1 mm for the QR24 method, we found that fewer than 20 positions were typically required by our method. This means that the QR36 method is especially suitable to handle cases where fewer tracking data are available.

Traditional hand-eye calibration method proposed by Shiu and Ahmad [23, 24] and Tsai and Lenz [25, 26] calculates the rotational and translational parts of the unknown matrices separately using matrix algebra. Li and Betsis applied a geometric approach and least-squares solution for hand-eye calibration [34]. Dual quaternion approach was also used for hand-eye calibration [35]. All those methods expect that orthogonal homogeneous matrices can be found. Moreover, a robust real-time hand-eye calibration method was proposed by Lars and Floris present [17]. A marker is attached to the robot’s third link for real-time hand-eye calibration. However, this method is not as precise as the QR24 algorithm. The total translational errors were 0.88 mm and 1.36 mm for QR24 method and real-time calibration method, respectively [17]. A robot system will not be calibrated perfectly, so orthogonality is not necessary. It is accepted or requested to permit non-orthogonality of the matrices in our calibration method. Our results demonstrated that the calibration method used in this study is more accurate than the classical hand-eye calibration approaches [23, 25]. In terms of translational accuracy, our method also outperforms the QR24 method. Therefore, the calibration method proposed in this study is more suitable for robotized transcranial magnetic stimulation.

We should point out that the maximum optimal translation errors for five handheld experiments per operator can reach 3 mm, as shown in Fig. 6a. This result is acceptable for a handheld navigated TMS experiment. During the robot-assisted TMS stimulation, the head motion is tracked using the marker on the subject’s head and compensated by the robot [18]. But, without robotic assistance and head motion compensation, the relative motion between the subject’s head and the handheld stimulation coil is greater than 3 mm during handheld TMS experiments [30]. Moreover, the calibration error of registration of the subject’s MRI and optical tracking device is also controlled at around 3 mm for handheld navigated TMS experiments [36]. However, for a robotic TMS system, which is designed for high-precision stimulation, the handheld coil calibration method is not suitable [37–39].

It is important to note that the new method has three limitations. It can only be applied when:

- It is accepted or requested to permit non-orthogonality of the matrices in our calibration method;
- The calibrated robotic TMS system is used in the same space where calibration was carried out;
- There are three unknown matrices that need to be solved in the robotic system.

## Conclusion

We have developed a new one-step calibration method to acquire three pose relationships from a navigated robotic system with a least-squares approach. The new method can significantly improve the accuracy of the robotic TMS system. Besides, the convergence speed is improved for the new method, which means that our method is particularly suited to handle fewer tracking data. The capability of the new method has been demonstrated for synchronous calibration and determination of the pose relationship of marker/end effector, probe/end effector, and robot/world for a robotic TMS system, which can be used to perform precision TMS experiment. Finally, for many robotic applications, where three unknown matrices need to be solved, the method presented in this paper provides an alternative solution to the classical approaches. In the future, it will be interesting to quantitatively compare the stimulation effect of robotic and manual techniques. Investigation on patients will then be pursued to evaluate the medical benefits of robotic TMS system.
